# Variant Discovery and Fine Mapping of Genetic Loci Associated with Blood Pressure Traits in Hispanics and African Americans

**DOI:** 10.1371/journal.pone.0164132

**Published:** 2016-10-13

**Authors:** Nora Franceschini, Cara L. Carty, Yingchang Lu, Ran Tao, Yun Ju Sung, Ani Manichaikul, Jeff Haessler, Myriam Fornage, Karen Schwander, Niha Zubair, Stephanie Bien, Lucia A. Hindorff, Xiuqing Guo, Suzette J. Bielinski, Georg Ehret, Joel D. Kaufman, Stephen S. Rich, Christopher S. Carlson, Erwin P. Bottinger, Kari E. North, D. C. Rao, Aravinda Chakravarti, Paula Q. Barrett, Ruth J. F. Loos, Steven Buyske, Charles Kooperberg

**Affiliations:** 1 Department of Epidemiology, University of North Carolina, Chapel Hill, North Carolina, United States of America; 2 Center for Translational Science, George Washington University and Children’s National Medical Center, Washington, District of Columbia, United States of America; 3 Genetics of Obesity and Related Metabolic Traits Program, The Charles Bronfman Institute for Personalized Medicine, Icahn School of Medicine at Mount Sinai, New York, New York, United States of America; 4 Department of Biostatistics, University of North Carolina, Chapel Hill, North Carolina, United States of America; 5 Division of Biostatistics, Washington University, St. Louis, Missouri, United States of America; 6 Center for Public Health Genomics, University of Virginia, Charlottesville, Virginia, 22908, United States of America; 7 Division of Public Health Sciences, Fred Hutchinson Cancer Research Center, Seattle, Washington, United States of America; 8 Brown Foundation Institute of Molecular Medicine & Human Genetics Center, University of Texas Health Science Center, Houston, Texas, United States of America; 9 Division of Genomic Medicine, National Human Genome Research Institute, National Institutes of Health, Bethesda, Maryland, United States of America; 10 Institute for Translational Genomics and Population Sciences and Department of Pediatrics, LABiomed at Harbor-University of California at Los Angeles Medical Center, Torrance, California, United States of America; 11 Division of Epidemiology, Department of Health Sciences Research, Mayo Clinic, Rochester, Minnesota, United States of America; 12 Center for Complex Disease Genomics, Johns Hopkins University School of Medicine, Baltimore, Maryland, United States of America; 13 Department of Specialties of Internal Medicine, Geneva University Hospital, Geneva, Switzerland; 14 Department of Environmental and Occupational Health Sciences, Epidemiology, and Medicine, University of Washington, Seattle, Washington, United States of America; 15 Department of Pharmacology, University of Virginia, Charlottesville, Virginia, United States of America; 16 Department of Statistics and Biostatistics, Rutgers University, Piscataway, New Jersey, United States of America; University at Buffalo—The State University of New York, UNITED STATES

## Abstract

Despite the substantial burden of hypertension in US minority populations, few genetic studies of blood pressure have been conducted in Hispanics and African Americans, and it is unclear whether many of the established loci identified in European-descent populations contribute to blood pressure variation in non-European descent populations. Using the Metabochip array, we sought to characterize the genetic architecture of previously identified blood pressure loci, and identify novel cardiometabolic variants related to systolic and diastolic blood pressure in a multi-ethnic US population including Hispanics (n = 19,706) and African Americans (n = 18,744). Several known blood pressure loci replicated in African Americans and Hispanics. Fourteen variants in three loci (*KCNK3*, *FGF5*, *ATXN2-SH2B3)* were significantly associated with blood pressure in Hispanics. The most significant diastolic blood pressure variant identified in our analysis, rs2586886/*KCNK3* (*P* = 5.2 x 10^−9^), also replicated in independent Hispanic and European-descent samples. African American and trans-ethnic meta-analysis data identified novel variants in the *FGF5*, *ULK4* and *HOXA-EVX1* loci, which have not been previously associated with blood pressure traits. Our identification and independent replication of variants in *KCNK3*, a gene implicated in primary hyperaldosteronism, as well as a variant in *HOTTIP* (*HOXA-EVX1*) suggest that further work to clarify the roles of these genes may be warranted. Overall, our findings suggest that loci identified in European descent populations also contribute to blood pressure variation in diverse populations including Hispanics and African Americans—populations that are understudied for hypertension genetic risk factors.

## Introduction

Blood pressure (BP) is an important predictor of health [1, 2], and has a lifetime impact on the risk of coronary heart disease, stroke, and kidney disease, which are conditions that disproportionately burden minority populations including Hispanic and African Americans [3]. The heritability of BP ranges from 30% to 55% [4–6]. Despite this moderate genetic contribution and the identification of a substantial number of genetic variants associated with BP traits, the vast majority of the heritability remains unexplained [6]. Genome-wide association studies (GWAS) have reported over 50 loci influencing BP and hypertension risk [7–12]. Most of these loci were identified in individuals of European ancestry [7–9]. Studies of East Asians [10, 13] and individuals with African ancestry [14, 15] have provided evidence for additional loci for BP, even though sample sizes were smaller than in studies of European ancestry individuals.

Hispanics are the largest growing minority in the US. Even though the prevalence of hypertension in this population is increasing, Hispanics are less likely to be aware of their high BP or seek treatment for hypertension [16, 17]. This racial/ethnic subgroup has also been underrepresented in GWAS of BP. Both Hispanic and African Americans are admixed populations and the allele frequency of variants and linkage disequilibrium (LD) patterns vary depending on the ancestral background of any individual. However, this diversity in genetic architecture can expedite the discovery of risk variants, as exemplified in a recent study that identify the *SLC16A11* locus associated with type 2 diabetes risk in Hispanics only [18].

Using the Metabochip array, we characterized and fine-mapped previously reported BP loci in a large multi-ethnic population. We also performed an array-wide analysis to identify novel cardiometabolic genetic variants (available on the Metabochip) associated with BP traits in Hispanic and African Americans. This study was performed as part of the Population Architecture using Genomics and Epidemiology (PAGE) Consortium [19] which was funded by the U.S. National Human Genome Research Institute to characterize GWAS-identified variants in ancestrally diverse populations.

## Materials and Methods

### Populations

This study includes self-reported Hispanic and African American adults with genotyped or imputed Metabochip genetic variants from the Population Architecture using Genomics and Epidemiology (PAGE) Study [19] cohorts and the Family Blood Pressure Program (FBPP) [20]. Samples from the Multi-Ethnic Study of Atherosclerosis (MESA) were used for replication. Study descriptions and methods for BP measurements are included in the Methods A in S1 File. All participants have given consent for genetic studies and data sharing and all studies have Institutional Research Board approval.

To generalize novel findings to individuals of European ancestry, we used summary data from the HapMap II meta-analysis of the International Consortium of Blood Pressure (ICBP), which included a discovery sample of 69,395 European-descent individuals [9]. Summary statistics from meta-analyses are publicly available and were downloaded from http://www.ncbi.nlm.nih.gov/projects/gap/cgi-bin/study.cgi?study_id=phs000585.v1.p1

### Genotyping and Outcomes

The Metabochip array design includes approximately 200,000 single nucleotide polymorphisms (SNPs) selected from GWAS loci as well as dense genotyping for fine-mapping of loci, as previously described [21]. Genotyping on PAGE samples was performed within PAGE [22]. Descriptions of the FBPP and replication study genotyping are summarized in the Methods A in S1 File. Standard QC filters were applied for samples and SNPs in PAGE: SNPs with call rates <95%, >2 replication errors, Hardy-Weinberg Equilibrium p-value<1 x 10^−6^, Illumina GenTrain Scores <0.6, Illumina Cluster Separation Scores <0.4 or discordant calls as specified in [22] were excluded. A subset of WHI African Americans had genome-wide data available from the SHARe GWAS; Metabochip genotypes were imputed in this sample, as described in Liu et al. [23]. Imputed genotypes with rsq<0.30 were excluded from analyses. Ancestry principal components were determined using Eigensoft software separately for each study [24]. Ancestry outliers and first-degree relatives were excluded for PAGE studies except for SOL, which adjusted for first degree relatives. Mt. Sinai Bio*Me* used genotypes from the Illumina HumanOmniExpress chip, which were imputed to 1000 Genome (1000G) Phase I integrated haplotype panels (March 2012) using IMPUTE2. Only SNPs with imputation quality> 0.4 and available on the Metabochip were examined in Mt. Sinai Bio*Me*.

All studies, except Mt. Sinai Bio*Me*, have standardized measures of BP, including sitting systolic and diastolic BP measured by trained personnel, according to a standardized protocol that specified a rest period of 5 minutes prior to measurement, bias-free instrumentation and timed, repeat measurements (Methods A in S1 File). Mt. Sinai Bio*Me* BP measures were extracted from electronic medical records (EMR). Hypertension was defined by a systolic BP > 140 and diastolic BP > 90 mm Hg or use of anti-hypertensive medication [25]. To correct for treatment effects, we added constants of 10 and 5 mm Hg to the systolic BP and diastolic BP, respectively, for individuals taking a BP lowering medication [7].

### Statistical analysis

Each study prepared race/ethnicity-specific summary models. Genotypes were coded using additive genetic models, or allelic dosage for imputed SNPs. For studies of unrelated individuals, we used linear regression models to test associations of genotypes with quantitative BP traits. For the studies that included related family members, we used methods to account for the correlation structure due to genetic relatedness. Specifically, in the FBPP studies, we used variance component models for quantitative traits and liability models for hypertension to account for the family-based study design. The HCHS/SOL has approximately 2000 related individuals, and a complex sampling design was used for recruitment. Therefore, we used the W-PS method proposed by Lin *et al*, which is a weighted version of generalized estimation equations, to account for unequal inclusion probabilities and family relationships[26]. All of these models were adjusted for age, sex (except WHI), body mass index (BMI) and principal components of ancestry.

Summary results for Hispanics from each study were combined for an inverse variance weighted fixed-effects meta-analysis of Hispanics only. Similarly, all African American results were combined for an inverse variance weighted fixed-effects meta-analysis of African Americans. Results from these race-specific meta-analyses are presented. We then conducted trans-ethnic meta-analyses of African Americans and Hispanics by combining all study-specific African American and Hispanic summary results from PAGE and the FBPP using (1) a fixed effects and (2) a random-effects approach described by Han and Eskin [27] as implemented in their software, METASOFT (http://genetics.cs.ucla.edu/meta). Between study/race-ethnicity effect sizes consistency was assessed using the Q test (Chi-squared p-value) and the I^2^ metric, where low I^2^ suggests little between-study/race-ethnicity variability. For the meta-analyses, we filtered out SNPs with a minor allele frequency less than 0.01. Results for all analyses were reported as Betas and standard errors (SE).

We sought to replicate our main findings from the discovery meta-analyses in MESA Hispanics and African Americans with the same protocols used in the discovery analysis (see Methods A in S1 File), and validated our findings in European ancestry samples using publically available ICBP data. A conservative SNP discovery significance threshold for the Metabochip array, *P* < 2.8 x 10^−7^ was used to account for the numbers of tests performed in the discovery analyses.

For our replication of previously reported GWAS SNPs (identified mainly in populations of European ancestry) in our multi-ethnic population, we defined nominally significant as ‘p<0.05 and not corrected for multiple testing.’

Given the different recruitment and BP measurement in the Mt. Sinai Bio*Me* sample, we performed a sensitivity analysis excluding the study from the Hispanic meta-analysis, and reviewed findings for consistency with the total Hispanic results. In a secondary analysis, we measured associations between hypertension and selected BP-associated SNPs using logistic regression models and reported odds ratios (OR) and 95% confidence intervals (95%CI).

We also fine-mapped 22 published BP loci previously identified in European and East Asian ancestry populations that were available on the Metabochip [Tables D-E in S1 File]. For these analyses, we focused on the African ancestry samples as this population, on average, has greater haplotype diversity and shorter LD blocks compared to Hispanics [28, 29]. In other words, a given SNP in a genomic locus of interest will typically have fewer numbers of SNPs highly correlated with it in African Americans, making it somewhat easier to reduce the size of a genomic locus of interest, and thus the number of potential causal SNP(s). To investigate SNPs in each BP-associated locus, we assessed whether the most significant SNP at each locus in African Americans was distinct from, but correlated (*r*^2^ ≥ 0.50) with the index (i.e. previously reported GWAS) SNP based on LD information from the 1000 Genomes Project (1000G) European ancestry populations (EUR). Using Locus Zoom plots, we also plotted other candidate functional variants in the region. The fine-mapping significance threshold was set at the Bonferroni-corrected threshold of *P*<0.05/number of Metabochip SNPs at each locus. Other LD estimates in the 1000G populations were obtained using the LDlink tool [30].

## Results

A total of 19,706 Hispanic individuals from three studies and 18,744 African American individuals from five studies contributed to the analyses (Table 1).

**Table 1:** Descriptive characteristics^a^ of the PAGE and FBBP studies by race/ethnicity.

Sample	Study	Total N	Female, %	Age, years	BMI, kg/m^2^	DBP, mmHg^b^	SBP, mmHg^b^	HTN Meds, %		HTN,%
**Hispanics**										
	WHI	5,155	100%	60 (7)	29 (6)	76 (10)	128 (19)	21%		38%
	HCHS/SOL	11,653	58%	47 (13)	30 (6)	75 (11)	125 (19)	17%		28%
	Mt. Sinai Bio*M*e	2,898	65%	49 (15)	29 (7)	74 (11)	123 (18)	28%		38%
**African Americans**										
	ARIC	3,340	63%	54 (6)	30 (6)	82 (13)	133 (23)	41%		55%
	CARDIA	1,797	58%	24 (4)	26 (6)	69 (10)	112 (11)	1%		1%
	WHI	11,800	100%	62 (7)	31 (7)	81 (10)	137 (20)	48%		61%
	HyperGEN									
	Cases	947	68% (68%)	51 (11)	33 (7)	76 (12)	135 (23)	92%		92%
	Controls	298	63% (63%)	35 (10)	31 (8)	70 (8)	116 (12)	0%		0%
	GenNet									
	Cases	153	54%	41 (7)	34 (10)	89 (14)	143 (18)	53%	53%	
	Controls	409	49%	35 (9)	29 (8)	71 (10)	117 (13)	0%	0%	

N, number; BMI, body mass index; DBP, diastolic blood pressure; SBP, systolic blood pressure; HTN, hypertension; Meds, medications

^**a**^Results presented as mean (standard deviation)

^**b**^Summaries based on BP values before correction for hypertension medication use

### Discovery and replication in Hispanics

In the discovery analysis, querying all SNPs on the Metabochip, there was little evidence for inflation due to population stratification in the Hispanic samples (Figure A in S1 File). Meta-analyses of the Hispanic studies identified 14 SNPs in three loci significantly associated with either systolic BP or diastolic BP (Table 2, and Tables A-B in S1 File). These SNPs were all in known BP loci rather than in Metabochip loci linked with other cardiometabolic traits. The most significant SNP (rs2586886/*G*) located in intron 1 of potassium channel, subfamily K, member 3 (*KCNK3*) on chromosome 12 was associated with a 0.72 mm Hg increase in diastolic BP (*P* = 5.2 x 10^−9^) (Table 2). This SNP was also associated with increased systolic BP (*P* = 3.8 x 10^−4^), and a 13% increased odds of hypertension (OR = 1.13; 95%CI: 1.06–1.22; *P* = 4.0 x 10^−4^) in 5822 hypertension cases and 13,477 controls. Other SNPs associated with BP in Hispanics were located at two previously established loci, *FGF5* (chromosome 4) and *ATXN2- SH2B3* (chromosome 12) (Tables A-B, and Figure B in S1 File). At *FGF5*, conditional analyses of the most significant SNP, rs1458038, which is also reported in individuals of European ancestry [31], supported the evidence for a single association at the locus (Table A in S1 File). At the *ATXN2-SH2B3* locus, the most significant SNP rs11065979 was in LD with a missense variant rs3184504 (minor allele frequency = 0.28, *P* = 1. 4 x 10^−7^, *r*^2^ = 0.91) (Table B and Figure B in S1 File) [9]. A nearby intronic SNP in *HECTD4* (rs11066188) also reached array-wide significance (*P* = 1.3 x 10^−7^) and was in LD with the aforementioned *ATXN2-SH2B3* variants (*r*^*2*^ = 0.74–0.84 in 1000G AMR).

**Table 2:** Main findings^a^ for meta-analysis of diastolic and systolic BP in PAGE Hispanic samples (n~19,706).

SNP	Chr	Gene Locus	Coded/ other allele	Coded allele frequency	Beta (SE)	*P*-value
**Diastolic BP**						
rs2586886	2	*KCNK3*	G/A	0.32	0.72(0.12)	5.2 x 10^−9^
rs1458038	4	*FGF5*	A/G	0.23	0.71(0.13)	1.2 x 10^−7^
rs11065979^b^	12	*SH2B3-ATXN2*	A/G	0.27	-0.73(0.13)	2.5 x 10^−8^
rs11066188^b^	12	*HECTD4*	A/G	0.26	0.70(0.13)	1.3 x 10^−7^
**Systolic BP**						
rs1458038	4	*FGF5*	A/G	0.23	1.37(0.22)	8.6 x 10^−10^

Chr, chromosome; SE, standard error

^a^Only the lowest p-value SNPs for each locus are shown.

^b^These SNPs are in linkage disequilibrium (r^2^ = 0.84 and D’ = 0.98) in the 1000G AMR population

The rs2586886/*KCNK3* SNP replicated in independent Hispanic samples from MESA for diastolic BP (*P* = 0.02, β = 0.78) (Table 3). This SNP is also significantly associated with systolic and diastolic BP in the European ancestry GWAS meta-analysis from the ICBP, and with systolic BP in African Americans, but was borderline significant for diastolic BP in African Americans (Table 2).

**Table 3:** Validation of the *KCNK3* rs2586886 associations with BP in independent multi-ethnic populations.

Study-race	BP Trait	Coded allele	Coded allele frequency	Beta(SE)	P value	Total N
MESA-Hispanics	Diastolic	G	0.30	0.94(0.37)	0.01	2,108
ICBP-whites	Diastolic	NA	0.33	NA	0.02	69,395
	Systolic	NA	0.33	NA	1.9 x 10^−3^	
PAGE & FBBP-	Diastolic	G	0.45	0.23(0.11)	0.04	18,744
African Americans	Systolic	G	0.45	0.42(0.18)	0.02	

BP, blood pressure; N, number; SE, standard error; ICBP, International Consortium of Blood Pressure; NA, not available in publicly available data

In sensitivity analyses excluding Mt. Sinai Bio*Me*, rs2586886/*KCNK3* remained significantly associated with diastolic BP, *P* = 3.1 x 10^−9^. *P*-values for other diastolic and systolic SNPs increased to values above the significance threshold, *P* = 2.8 x 10^−7^. However, none of the SNPs showed evidence of heterogeneity by study in the meta-analysis of all Hispanics including Mt. Sinai, all heterogeneity *P*>0.4 and I^2^~0. Using PAGE Hispanic results, we performed a look-up of previously identified GWAS SNPs in 22 loci (Table C in S1 File). GWAS SNPs from 15 loci replicated in Hispanics at p<0.05.

### Replication, fine mapping and discovery in African Americans

We first attempted to replicate the 22 BP loci previously identified in GWAS of BP in European ancestries using African American samples. These meta-analyses identified SNPs in *FGF5*, *ATP2B1*, *TBX3* and *LOC339593* nominally associated with systolic BP (Table D in S1 File), and SNPs in *FGF5*, *SLC39A8*, *ARHGAP42*, *ADM*, *CPLX3*, *LOC339593* and *ZNF831* nominally associated with diastolic BP, all *P* <0.05 (Table E in S1 File). To fully capture the European-ancestry GWAS SNP ‘signal’, we then extended the association analyses in African Americans to include all SNPs in modest LD (r^2^≥0.5 in 1000G EUR) with each reported GWAS SNP. These analyses identified additional associations in African Americans at *MTHFR*, *EBF1*, *C10orf107*, *CYP17A1*, *NT5C2*, *CSK*, and *PLCD3* for either diastolic or systolic BP with *P*-values ranging from 6.9 x 10^−5^ to 0.04, (Tables D-E in S1 File).

Rs56153133/*CLCN6* was significantly associated with diastolic BP (*P* = 6.9 x 10^−5^) at the locus-level significance threshold (*P*<1.4 x 10^−4^) in African Americans (Figure C in S1 File). This SNP is in high LD (*r*^2^ = 0.98 in 1000G EUR) with the previously reported GWAS SNP rs17367504 in the *MTHFR* locus. However, rs17367504/*MTHFR* was not associated with diastolic BP in African Americans (*P* = 0.24), in spite of its relatively high correlation with rs56153133 (*r*^2^ = 0.65 in PAGE African Americans). In a model conditioning on rs17367504, rs56153133 remained significantly associated with diastolic BP, *P* = 1.3x10^-6^, providing evidence to support its independence from rs17367504. However, the rs56153133-diastolic BP association did not replicate in the independent, though smaller (n = 2550) MESA African American sample, *P* = 0.88.

The strongest associations in the systolic BP fine mapping were located in the 15q26.1 locus (*FURIN*), but none of the SNPs reached the fine-mapping significance threshold for the locus (*P*<5 x 10^−4^). The most significant (*P* = 8.0 x 10^−4^) SNP in this locus in African Americans is rs6224, an intronic variant in *FURIN*. Rs6224/*FURIN*, is in low LD (*r*^2^ = 0.08) in African Americans with the prior GWAS SNP in this region, rs2521501/*FES*, and in modest LD (*r*^2^ = 0.46) with it in the 1000G EUR (Figure D in S1 File).

In a full Metabochip scan testing for new SNP associations with BP traits in African Americans, no SNPs reached the discovery (multiple testing-adjusted) significance threshold (*P*<2.8 x 10^−7^).

### Trans-ethnic meta-analyses

To increase power to identify associations in the discovery scan, we also performed trans-ethnic meta-analyses by combining results from Hispanics and African Americans. We used two approaches for the meta-analyses: a fixed-effects model, and a random-effects model optimized to detect associations under heterogeneity, as implemented in MetaSoft [27]. While no novel loci were identified, there were eight array-wide significant (*P*<2.8 x 10^−7^) SNPs in *ULK4*, and two SNPs in 5`region of *FGF5* associated with diastolic BP (Table F in S1 File).

Several SNPs were also significantly associated with systolic BP including rs2023843, a novel intronic SNP in *HOTTIP* (in the *HOXA-EVX1* locus) (Figure E in S1 File). This SNP replicated in MESA Hispanics, *P* = 0.038 and was borderline significant in MESA African Americans, *P* = 0.064. Rs2023843 is in modest LD (r^2^ = 0.27 in 1000G AFR) with rs11564022/ *HOXA-EVX1* previously reported in African Americans (and not available on the Metabochip), though LD with it in 1000G AMR Hispanics is very low (r^2^ = 0.03). SNPs upstream of *FGF5* were also significantly associated with systolic BP in the trans-ethnic analysis, and most appear to be mainly driven by the Hispanic American results. The most significant trans-ethnic SNP at the *FGF5* locus, rs13125101 is in high LD (*r*^2^>0.8 in CEU) with the prior GWAS SNPs (rs16998073 and rs11099098) suggesting that it may reflect the same signal (Figure F in S1 File). While this locus is not novel, we identified a significant *FGF5* intronic SNP not previously associated with BP, rs36034102. However this SNP was not associated with systolic BP (*P* = 0.25) in the smaller sample of MESA Hispanics (n = 2106) although the direction and magnitude of the beta coefficient were similar, β = 0.89 in MESA, β = 1.05 in PAGE (data not shown).

All SNPs significant in the fixed-effects trans-ethnic meta-analyses were also significant when using the Han and Eskin (HE) random-effects meta-analysis models with the exception of one systolic BP SNP (rs72656599/5’ of *FGF5*), which was borderline significant in the random-effects model. As expected, *P*-values for the two models were generally comparable, with fixed effects models having lower *P*-values, except in the presence of high heterogeneity (I^2^≥ 80) between the race/ethnicity groups. In cases of high heterogeneity, the HE random-effects *P*-values were lower than those from fixed effects models.

## Discussion

We describe the replication of several BP loci in two lesser studied populations, US Hispanics and African Americans and also report novel SNPs associations in these previously published loci. In Hispanics, we identified 14 variants in known loci associated with blood pressure, including an intronic variant in *KCNK3* associated with diastolic BP. In trans-ethnic analyses, we also identified a variant in *HOTTIP* associated with systolic BP. Both of these variants replicated in an independent sample of Hispanics.

In addition to diastolic BP, the *KCNK3* intronic SNP was also significantly associated with systolic BP and associations replicated in European and African ancestry samples. Each copy of the *KCNK3* rs2586886-*G* allele was associated with 13% increased odds of hypertension in Hispanics. *KCNK3* encodes TASK-1, a member of the superfamily of potassium channel proteins, which functions primarily to control the resting membrane potential in many cell types. *KCNK3* has been previously associated with mean arterial pressure and systolic blood pressure in GWAS of individuals of European and East Asian ancestry, and more recently in candidate gene analyses of African Americans and Hispanics [32–34]. The previously reported association is for a SNP located at 2kb upstream of *KCNK3* (rs1275988) [32, 33], which is in LD with rs2586886 in our Hispanic sample (r^2^ = 0.70) and in 1000G EUR (r^2^ = 0.94), and thus likely represent the same association. Using 1000G AMR haplotypes, two SNPs (rs12476527/promoter and rs12775923/first intron) fall within putative regulatory elements that serve as binding sites for transcription factors and impact transcription factor binding in relevant tissues, suggesting a possible mechanism for the BP association at this locus (Table G in S1 File shows sources). Both of these SNPs are in strong LD with our identified SNP rs2586886, with r^2^ = 0.69 and 0.79, respectively, in the 1000G AMR population. The observation that rs1275988 is enriched with DNA methylation sites lends further support to the notion that regulatory pathways contribute to BP variation [33].

*KCNK3* protein TASK-1 is highly expressed in the human adrenal cortex in the aldosterone-synthesizing layer, the zona glomerulosa, that regulates aldosterone secretion [35]. Female TASK1-/- mice lacking *KCNK3* develop hypokalemia and low-renin hypertension that is normalized by mineralocorticoid receptor blockade but controlled by ACTH, a consequence of misallocation of aldosterone synthase in the zona fasciculate [36]. TASK-1 (*kcnk3*) and TASK-3 (*kcnk9*) double-knockout mice (TASK-1^-/-^/TASK-3^-/-^) recapitulate the key features of human primary hyperaldosteronism [37], the most common cause of endocrine hypertension. Specifically, they have elevated aldosterone production despite low plasma renin, and increased aldosterone levels on low-sodium diets that is not normalized by angiotensin-2 receptor blockade, nor suppressed by a high sodium loading. Our *KCNK3* SNP, rs2586886, has been recently shown to associate with aldosterone levels by our collaborators, although findings did not replicate in an independent sample and may be confounded by use of medications that suppress aldosterone[34]. Overall, our findings that the common *KCNK3* variant is associated with hypertension in Hispanics suggest that further work is needed to explore hypertension related salt-sensitivity in this population. These findings may also have clinical implications regarding the choice of medications for hypertension treatment in this population, which should be explored in future studies.

We replicated several GWAS loci in our population, but not necessarily the previously reported GWAS SNPs. These include the *FGF5* and *ATXN2- SH2B3* loci in Hispanics and *CLCN6/MTHFR* locus in African Americans. In total, 16 of the 22 BP loci tested nominally replicated in African Americans and GWAS SNPs in 15 of 22 loci nominally replicated in Hispanics. Fine-mapping of the *CLCN6/MTHFR* and *FES/FURIN* loci identified potential causal variants, though these findings should be interpreted cautiously given their lack of robust replication and/or modest *P*-values.

Although the trans-ethnic meta-analysis did not reveal new BP loci, we did identify novel SNPs not previously associated with BP traits in loci identified in European-ancestry individuals, including the *FGF5* [8], *ULK4* [38] and the *HOXA-EVX1* loci. SNP rs2023843 is an intronic variant of *HOTTIP* at the *HOXA-EVX1* locus. Three SNPs in this locus associated with BP traits (rs17428471, rs17471520, and rs11564022) were recently identified in individuals of African ancestry [15] (including a subset of our population). LD between rs2023843/*HOTTIP* and these variants is very low (r^2^<0.05) in Hispanics and African American 1000G populations with the exception of rs11564022, in which r^2^ = 0.27 in African Americans. Rs11564022 was not available in large numbers of our sample, so were unable to test the independence of the *HOTTIP* association.

In general, we observed less significant findings when the Mt. Sinai Bio*Me* sample (~15% of the total sample) was excluded from analyses in Hispanics. Discordance between clinic measurements, such as those obtained in the Mt.Sinai Bio*Me* sample, and research BP measurements has been reported to either under- or overestimate BP [39], potentially increasing measurement error and reducing power for SNP associations. Notably, the rs2586886/*KCNK3* association in Hispanics was more robust after exclusion of the Mt.Sinai Bio*Me* sample. An additional key limitation of our study was that fine-mapping could only be performed in Metabochip regions with high density genotyping. For example, we were unable to fine-map the *ULK4* or *HOTTIP* (*HOXA-EVX*) loci due to low density genotyping in these regions on the Metabochip. Furthermore, BP loci for replication and novel SNPs for discovery were limited to SNPs available on the Metabochip array.

In summary, in this first large array scan of BP traits in Hispanics, we identified *KCNK3* and *HOTTIP (HOXA-EVX1)* as important loci associated with BP variation in Hispanics and African American populations, respectively. We also replicated several BP loci identified in individuals of European ancestry in both Hispanics and African Americans supporting the importance of many common BP genetic loci across diverse populations. Our work demonstrates how studying genetically diverse populations may reveal novel genetic variants that can be used to better refine the location of causal variant(s), and inform research of potential drug targets in affected populations.

## Supporting Information

S1 File Discovery and fine mapping of genetic loci associated with blood pressure traits in Hispanics and African Americans. Q-Q plots of Hispanic Study-Specific Results for Systolic and Diastolic Blood Pressure (Figure A). Regional plots for *SH2B3* (A) and *TRAFD1* (B) loci for diastolic BP in Hispanics (Figure B). Fine-mapping of the *MTHFR/CLCN6* region in African Americans for Diastolic Blood Pressure (Figure C). Fine-mapping regional plots of 15q26.1 SBP Locus in African Americans (Figure D). Trans-ethnic results: fine-mapping regional plots of the SBP *HOTTIP* Locus (Figure E). Trans-ethnic results: fine mapping regional plots of the SBP *FGF5* Locus (Figure F). Significant SNPs at the *FGF5* locus for Diastolic and Systolic BP in Hispanics (Table A). Significant SNPs in the *SH2B3* locus for Diastolic BP in Hispanics (Table B). Replication of BP Trait GWAS SNPs for Blood Pressure Traits in Meta-analyses of Hispanics from PAGE (Table C). Replication and Fine Mapping of BP Trait GWAS SNPs for Systolic BP in African Americans from PAGE and FBPP (Table D). Replication and Fine Mapping of BP Trait GWAS SNPs for Diastolic BP in African Americans from PAGE and FBPP (Table E). Significant Results from the Trans-ethnic Meta-analysis of Blood Pressure Traits (Table F). Annotation Database/Tool and Websites (Table G). Study Description and Blood Pressure Measurements (Methods A).
(PDF)
