# Supplementary material for: Variant Discovery and Fine Mapping of Genetic Loci Associated with Blood Pressure Traits in Hispanics and African Americans
Source: PLoS One. 2016 Oct 13;11(10):e0164132. doi: 10.1371/journal.pone.0164132 (PMC5063457; doi:10.1371/journal.pone.0164132)
Supplement: S1 File — Q-Q plots of Hispanic Study-Specific Results for Systolic and Diastolic Blood Pressure (Figure A). Regional plots for SH2B3 (A) and TRAFD1 (B) loci for diastolic BP in Hispanics (Figure B). Fine-mapping of the MTHFR/CLCN6 region in African Americans for Diastolic Blood Pressure (Figure C). Fine-mapping regional plots of 15q26.1 SBP Locus in African Americans (Figure D). Trans-ethnic results: fine-mapping regional plots of the SBP HOTTIP Locus (Figure E). Trans-ethnic results: fine mapping regional plots of the SBP FGF5 Locus (Figure F). Significant SNPs at the FGF5 locus for Diastolic and Systolic BP in Hispanics (Table A). Significant SNPs in the SH2B3 locus for Diastolic BP in Hispanics (Table B). Replication of BP Trait GWAS SNPs for Blood Pressure Traits in Meta-analyses of Hispanics from PAGE (Table C). Replication and Fine Mapping of BP Trait GWAS SNPs for Systolic BP in African Americans from PAGE and FBPP (Table D). Replication and Fine Mapping of BP Trait GWAS SNPs for Diastolic BP in African Americans from PAGE and FBPP (Table E). Significant Results from the Trans-ethnic Meta-analysis of Blood Pressure Traits (Table F). Annotation Database/Tool and Websites (Table G). Study Description and Blood Pressure Measurements (Methods A). (PDF) [file pone.0164132.s001.pdf]

Supporting Information: Discovery and fine mapping of genetic loci associated with blood pressure traits in Hispanics and African Americans

Supplemental Tables

Table A. Significant SNPs at the *FGF5* locus for Diastolic and Systolic BP in Hispanics

SNP	Coded allele	Other allele	Coded allele frequency	Beta	SE	*P*-value
**Diastolic BP**						
rs1458038	A	G	0.23	0.71	0.13	1.2×10^{-7}
rs16998073	A	T	0.24	0.69	0.13	1.7×10^{-7}
**Systolic BP**						
rs1458038	A	G	0.23	1.37	0.22	8.6×10^{-10}
rs11099098	A	C	0.23	1.37	0.23	1.1×10^{-9}
rs13125101	A	G	0.24	1.34	0.22	1.4×10^{-9}
rs16998073	A	T	0.24	1.32	0.22	2.3×10^{-9}
rs36034102	A	C	0.25	1.19	0.22	4.4×10^{-8}
rs10857147	A	T	0.26	1.14	0.22	1.2×10^{-7}
rs72656599	A	G	0.25	1.14	0.22	1.9×10^{-7}

SNPs with $P < 2.8 \times 10^{-7}$ are shown.

Table B. Significant SNPs in the *SH2B3* locus for Diastolic BP in Hispanics (see also Figure B)

SNP	Function	Coded allele	Other allele	Frequency coded allele	beta	SE	*P*	Total N
rs11065987		A	G	0.74	-0.72	0.13	4.6×10^{-8}	19,703
rs17630235	intron	A	G	0.26	0.70	0.13	1.2×10^{-7}	19,703
rs11066188	intron	A	G	0.26	0.70	0.13	1.3×10^{-7}	19,706
rs3184504	missense	A	G	0.28	0.68	0.13	1.4×10^{-7}	19,691

SNPs with $P < 2.8 \times 10^{-7}$ are shown. (see also Figure B)

Table C. Replication of BP Trait GWAS SNPs for Blood Pressure Traits in Meta-analyses of Hispanics from PAGE

					PAGE Hispanics	Diastolic BP Results in PAGE Hispanics		Systolic BP Results in PAGE Hispanics	
**Chr**	**Gene(s)**	**GWAS SNP**	**GWAS Locus[a]**	**Ref**	**CA/ CAF**	**Beta (SE) in mmHg**	**GWAS SNP *P* -value**	**Beta (SE) in mmHg**	**GWAS SNP *P* -value**
1	*MTHFR*/intron	rs17367504	1	1	A/0.90	0.51 (0.19)	0.0066	0.77 (0.31)	0.0118
3	*MECOM*/intron	rs1918974	2	1	A/0.64	-0.28 (0.12)	0.0202		NS
	MECOM/intron	rs448378	2	2	A/0.60	-0.27 (0.12)	0.0200		NS
4	5’ of *FGF5*	rs16998073	3	1	A/0.24	0.69 (0.13)	1.7 x10^{-7}		2.3 x10^{-9}
	5’ of *FGF5*	rs1458038	3	3	A/0.23	0.71 (0.13)	1.2 x10^{-7}	1.37 (0.22)	8.6 x10^{-10}
	SLC39A8/missense	rs13107325	4	3	A/0.05	-0.74 (0.27)	0.0061		NS
5	3’ of *NPR3*	rs1173771	5	4	A/0.39	-0.35 (0.12)	0.0022	-0.54 (0.19)	0.0049
	3’ of *EBF1*	rs9313772	6	4	A/0.34	-0.29 (0.12)	0.0138	-0.52 (0.2)	0.0089
6	*HFE*/missense	rs1799945	7	3			NS		NS
	3’ of *HIST1H1T*	rs198846	7	4			NS		NS
10	*C10orf107*/intron	rs1530440	8	1			NS		NS
	C10orf107/intron	rs4590817	9	1			NS		NS
	CYP17A1/intron	rs1004467	10	2	A/0.83	0.66 (0.15)	1.5x10^{-5}	1.15 (0.25)	6.4 x10^{-6}
	NT5C2/3'-UTR	rs11191548	10	1	A/0.86	0.61 (0.17)	0.0003	1.17 (0.28)	2.8 x10^{-5}
	PLCE1/intron	rs9663362	11	4	C/0.58	0.26 (0.12)	0.0246	0.39 (0.19)	0.0448
	PLCE1/intron	rs932764	11	3			NS		NS
11	*ADM*/intron	rs7129220	12	4			NS		NS
	PLEKHA7/intron	rs381815	13	2			NS		NS
	PLEKHA7/intron	rs11024074	13	2	A/0.71	-0.27 (0.12)	0.0301	-0.45 (0.21)	0.0281
	ARHGAP42/intron	rs633185	14	4	C/0.62		NS	0.46 (0.20)	0.0197
12	*ATP2B1*/intron	rs2681472	15	2	A/0.87	0.70 (0.17)	4.1x10^{-5}	1.07 (0.29)	0.0002
	SH2B3/missense	rs3184504	16	2	A/0.28	0.68 (0.13)	1.4x10^{-7}	0.67 (0.21)	0.0017
	ATXN2/intron	rs653178	16	1	A/0.72	-0.66 (0.13)	3.2x10^{-7}	-0.68 (0.21)	0.0014
	5’ of *TBX3*	rs2384550	17	2			NS		NS

15	*CSK*/intron	rs1378942	18	1	A/0.38	-0.3 (0.12)	0.0137	-0.58 (0.20)	0.0040
	3’ of *CPLX3*	rs6495122	18	2	A/0.56	0.3 (0.11)	0.0092	0.37 (0.19)	0.0488
	FES/intron	rs2521501	19	4	A/0.22	0.31 (0.14)	0.0282		NS
17	*PLCD3*/intron	rs12946454	20	1			NS		NS
20	5’ of *ZNF831*	rs6015450	21	3			NS		NS
	3' of *LOC339593*	rs1327235	22	3	A/0.67	-0.43 (0.12)	0.0005	-0.60 (0.20)	0.0031

Ref= reference, CA= coded allele, CAF= coded allele frequency, NS=not significant (p≥0.05)

[a]number of independent GWAS loci tested, i.e. indicating if the Metabochip GWAS SNPs on same chromosome are in modest linkage equilibrium, (LD<0.5 in 1000G EUR)

Table D. Replication and Fine Mapping of BP Trait GWAS SNPs for Systolic BP in African Americans from PAGE and FBPP

					GWAS SNP Replication for Systolic BP			Fine Mapping of GWAS Locus					
Chr	Gene(s)	GWAS SNP	GWAS Locus[a]	Ref	CA/CAF	Beta (SE)	GWAS SNP *P*-value	# of SNPs in LD[b]	Most sig SNP in Locus[c]	CA/CAF	Beta(SE)	Top SNP *P*-value	r^2 with GWAS SNP in EUR[d]
1	*MTHFR*/intron	rs17367504	1	[1]			NS	61	rs6687229	A/0.10	-0.8 (0.3)	0.0200	0.67
3	*MECOM*/intron	rs1918974	2	[1]			NS	29					
	MECOM/intron	rs448378	2	[2]			NS	29					
4	5' of *FGF5*	rs16998073	3	[1]	A/0.11	0.59 (0.29)	0.04	17	rs36034102	A/0.10	0.8 (0.3)	0.0137	0.77
	5' of FGF5	rs1458038	3	[3]			NS	15	rs36034102	A/0.10	0.8 (0.3)	0.0137	0.73
	SLC39A8/missense	rs13107325	4	[3]			NS	0					
5	3' of *NPR3*	rs1173771	5	[4]			NS	8					
	3' of *EBF1*	rs9313772	6	[4]			NS	114	rs10042219	A/0.58	-0.5 (0.2)	0.0070	0.69
6	*HFE*/missense	rs1799945	7	[3]			NS	20					
	3' of *HIST1H1T*	rs198846	7	[4]			NS	21					
10	*C10orf107*/intron	rs1530440	8	[1]			NS	39					
	C10orf107/intron	rs4590817	9	[1]			NS	30	rs61850467	A/0.89	0.6 (0.3)	0.0468	0.97
	CYP17A1/intron	rs1004467	10	[2]			NS	61	rs3740393	C/0.84	0.7 (0.3)	0.0061	0.54
	NT5C2/3'-UTR	rs11191548	10	[1]			NS	61	rs3740393	C/0.84	0.7 (0.3)	0.0061	0.55
	PLCE1/intron	rs9663362	11	[4]			NS	12					
	PLCE1/intron	rs932764	11	[3]			NS	12					
11	ADM/intron	rs7129220	12	[4]			NS	123	rs72851674	C/0.02	1.4 (0.6)	0.0196	0.97
	PLEKHA7/intron	rs11024074	13	[2]			NS	4					
	PLEKHA7/intron	rs381815	13	[2]			NS	4					
	ARHGAP42/intron	rs633185	14	[4]			NS	18	rs667575	A/0.89	-0.6 (0.3)	0.0261	0.82
12	*ATP2B1*/intron	rs2681472	15	[2]	A/0.90	0.74 (0.30)	0.012	4	rs11105383	A/0.88	0.8 (0.3)	0.0028	0.50
	SH2B3/missense	rs3184504	16	[2]			NS	6					

	ATXN2/intron	rs653178	16	1			NS	6					
	5' of TBX3	rs2384550	17	2	A/0.33	-0.59 (0.19)	0.002	18	rs7961916	A0.40	-0.7 (0.2)	0.0005	0.60
15	*CSK*/ intron	rs1378942	18	1			NS	59					
	3' of *CPLX3*	rs6495122	18	2			NS	49					
	FES/intron	rs2521501	19	4			NS	11					
17	*PLCD3*/intron	rs12946454	20	1			NS	11	rs9894577	A/0.31	-0.5 (0.2)	0.0200	0.60
20	5' of *ZNF831*	rs6015450	21	3			NS	5					
	3' of LOC339593	rs1327235	22	3	T/0.50	-0.45 (0.18)	0.013	7	rs1887320	A/0.50	0.5 (0.2)	0.0125	1.0

Ref= reference, CA= coded allele, CAF= coded allele frequency, LD=linkage disequilibrium, NS=not significant ($p \geq 0.05$)

[a]number of independent GWAS loci tested, i.e. indicating if the Metabochip GWAS SNPs on same chromosome are in modest linkage equilibrium, (LD<0.5 in 1000G EUR)

[b]based on $r^2 \geq 0.50$ in 1000G European (EUR) populations (Phase 3)

[c]only SNPs with $P<0.05$ are shown

[d]in 1000G European (EUR) populations (Phase 3)

Table E. Replication and Fine Mapping of BP Trait GWAS SNPs for Diastolic BP in African Americans from PAGE and FBPP

					**GWAS SNP Replication for Diastolic BP in African Americans**			**Fine Mapping of GWAS Locus in African Americans**					
**Chr**	**Gene(s)**	**GWAS SNP**	**GWAS Locus[a]**	**Ref**	**CA/ CAF**	**Beta (SE)**	**GWAS SNP *P* -value**	**# of SNPs in LD[b]**	**Most sig SNP in Locus[c]**	**CA/CAF**	**Beta(SE)**	**Top SNP *P*-value**	**r^2 with GWAS SNP in EUR[d]**
1	*MTHFR*/intron	rs17367504	1	1			NS	61	rs56153133	A/0.88	0.7(0.2)	6.92E-05	0.97
3	*MECOM*/intron	rs1918974	2	1			NS	29					
	MECOM/intron	rs448378	2	2			NS	29					
4	5' of *FGF5*	rs16998073	3	1			NS	17	rs1902859	A/0.88	-0.4(0.2)	0.0269	0.63
	5' of *FGF5*	rs1458038	3	3			NS	15	rs1902859	A/0.88	-0.4(0.2)	0.0269	0.63
	SLC39A8/missense	rs13107325	4	3	T/0.01	-0.96 (0.47)	0.04	0					
5	3' of *NPR3*	rs1173771	5	4			NS	114					
	3' of *EBF1*	rs9313772	6	4			NS	8	rs12187534	A/0.93	0.7(0.2)	0.0025	0.86
6	*HFE*/missense	rs1799945	7	3			NS	20					
	3' of *HIST1H1T*	rs198846	7	4			NS	21					
10	*C10orf107*/intron	rs1530440	8	1			NS	39	rs72831369	A/0.04	-0.6 (0.3)	0.0414	0.61
	C10orf107/intron	rs4590817	9	1			NS	30	rs61850467	A/0.90	0.4 (0.2)	0.0408	0.97
	CYP17A1/intron	rs1004467	10	2			NS	61					
	NT5C2/3'-UTR	rs11191548	10	1			NS	61					
	PLCE1/intron	rs9663362	11	4			NS	12					
	PLCE1/intron	rs932764	11	3			NS	12					
11	*ADM*/intron	rs7129220	12	4	T/0.08	0.47 (0.20)	0.02	123	rs1450276	A/0.10	0.4 (0.2)	0.0201	0.91
	PLEKHA7/intron	rs381815	13	2			NS	4					
	PLEKHA7/intron	rs11024074	13	2			NS	4					
	ARHGAP42/intron	rs633185	14	4	C/0.80	0.28	0.04	18					

						(0.14)							
12	*ATP2B1*/intron	rs2681472	15	2			NS	4					
	SH2B3/missense	rs3184504	16	2			NS	6					
	ATXN2/intron	rs653178	16	1			NS	6					
	5’ of *TBX3*	rs2384550	17	2			NS	18	rs7977406	T/0.40	-0.3(0.1)	0.0075	0.64
15	*CSK*/intron	rs1378942	18	1			NS	59	rs6495122	A/0.72	0.3(0.1)	0.0083	0.61
	3’ of *CPLX3*	rs6495122	18	2	A/0.72	0.33 (0.12)	0.008	49					
	FES/intron	rs2521501	19	4			NS	11					
17	*PLCD3*/intron	rs12946454	20	1			NS	11					
20	5’ of *ZNF831*	rs6015450	21	3	A/0.81	-0.31 (0.14)	0.03	5					
	3' of LOC339593	rs1327235	22	3	T/0.49	-0.32 (0.11)	0.004	7	rs1887320	A/0.51	0.32(0.11)	0.0031	1.0

Ref= reference, CA= coded allele, CAF= coded allele frequency, LD=linkage disequilibrium

[a]number of independent GWAS loci tested, i.e. indicating if the Metabochip GWAS SNPs on same chromosome are in modest linkage equilibrium, (LD<0.5 in 1000G EUR)

[b]based on $r^2 \geq 0.50$ in 1000G European (EUR) populations (Phase 3)

[c]only SNPs with P<0.05 are shown

[d]in 1000G European (EUR) populations (Phase 3)

Table F. Significant[a] Results from the Trans-ethnic Meta-analysis of Blood Pressure Traits

Trait	SNP	Chr: annotation	Hispanics *p* -value	African Americans *p* -value	FE *p* -value[b]	HE-RE *p* –value[c]	I^2
DBP	rs2586886	2: intron *KCNK3*	5.2 $x10^{-9}$	3.5 $x10^{-2}$	4.6 $x10^{-8}$	9.5 $x10^{-9}$	89
	rs2272007	3: missense *ULK4*	1.4 $x10^{-4}$	4.2 $x10^{-5}$	2.3 $x10^{-8}$	3.2 $x10^{-8}$	0
	rs7626217	3: intron *ULK4*	4.2 $x10^{-4}$	5.9 $x10^{-5}$	9.3 $x10^{-8}$	1.1 $x10^{-7}$	0
	rs1716975	3: missense *ULK4*	6.3 $x10^{-4}$	4.1 $x10^{-5}$	9.5 $x10^{-8}$	1.1 $x10^{-7}$	0
	rs1016669	3: intron *ULK4*	4.3 $x10^{-4}$	6.9 $x10^{-5}$	1.1 $x10^{-7}$	1.3 $x10^{-7}$	0
	rs6599176	3: intron *ULK4*	2.6 $x10^{-3}$	1.2 $x10^{-5}$	1.2 $x10^{-7}$	1.5 $x10^{-7}$	0
	rs9874975	3: intron *ULK4*	8.7 $x10^{-4}$	4.3 $x10^{-5}$	1.4 $x10^{-7}$	1.6 $x10^{-7}$	0
	rs6599178	3: intron *ULK4*	2.1 $x10^{-4}$	1.8 $x10^{-4}$	1.4 $x10^{-7}$	1.7 $x10^{-7}$	0
	rs1717017	3: intron *ULK4*	3.7 $x10^{-4}$	1.5 $x10^{-4}$	2.1 $x10^{-7}$	2.5 $x10^{-7}$	0
	rs1458038	4: 5` of *FGF5*	1.2 $x10^{-7}$	7.8 $x10^{-2}$	6.6 $x10^{-8}$	7.5 $x10^{-8}$	2
	rs16998073	4: 5` of *FGF5*	1.7 $x10^{-7}$	8.5 $x10^{-2}$	1.9 $x10^{-7}$	2.1 $x10^{-7}$	69
SBP	rs13125101	4: 5` of *FGF5*	1.4 $x10^{-9}$	3.0 $x10^{-1}$	8.6 $x10^{-10}$	1.3$x10^{-9}$	0
	rs16998073	4: 5` of *FGF5*	2.3 $x10^{-9}$	4.2 $x10^{-2}$	2.3 $x10^{-9}$	2.5 $x10^{-9}$	76
	rs11099098	4: 5` of *FGF5*	1.1 $x10^{-9}$	1.6 $x10^{-1}$	3.6 $x10^{-9}$	3.9 $x10^{-9}$	77
	rs36034102	4: intron *FGF5*	4.4 $x10^{-8}$	1.4 $x10^{-2}$	3.8 $x10^{-9}$	5.5 $x10^{-9}$	23
	rs1458038	4: 5` of *FGF5*	8.6 $x10^{-10}$	2.3 $x10^{-1}$	7.8 $x10^{-9}$	5.3 $x10^{-9}$	83
	rs72656599	4: 5` of *FGF5*	1.9$x10^{-7}$	1.4$x10^{-1}$	2.4$x10^{-7}$	2.9$x10^{-7}$	61
	rs2023843	7: intron *HOTTIP*	1.1 $x10^{-3}$	4.2 $x10^{-5}$	2.2 $x10^{-7}$	2.7 $x10^{-7}$	0

CHR, chromosome; FE, fixed effects; RE, random effects; I^2, statistic describing the percentage of variation across samples (in the meta-analysis) that is due to heterogeneity rather than chance

[a]Significant (less than the scan-wide threshold of $p<2.8 \times 10^{-7}$) for the fixed effects model

[b]fixed effects meta-analysis *p* -value

[c]revised random effects model (Han and Eskin, AJHG 2011)

Table G. Annotation Database/Tool and Websites

Annotation Database/Tool	Website
Haploreg	http://www.broadinstitute.org/mammals/haploreg
UCSC Genome Browser	http://genome.ucsc.edu
**Annotations**	**Website/Reference**
**Open Chromatin (DHS) – 300 cell types/tissues**	
ENCODE	http://genome.ucsc.edu/cgi-bin/hgHubConnect
Roadmap Epigenomics	http://vizhub.wustl.edu/VizHub/RoadmapReleaseAll.txt
**Transcription Factor Binding**	
ENCODE TFBS ChIPseq	http://genome.ucsc.edu/cgi-bin/hgHubConnect
JASPAR	http://jaspar.genereg.net/
CONSITE	http://consite.genereg.net/
Haploreg	http://www.broadinstitute.org/mammals/haploreg
Transfac	http://www.gene-regulation.com/pub/databases.html
**Chromatin marks**	
Integrative chromatin state	ENCODE and Roadmap (PMID: 23221638, 22955616)
Splice Site	
BDGP	http://www.fruitfly.org/
SPANR	Splice variant disruptions http://tools.genes.toronto.edu/
**Functional Segmentation**	
ChromHmm	http://compbio.mit.edu/ChromHMM/
Segway	http://noble.gs.washington.edu/proj/segway/

Supplementary Functional Annotation description

To further prioritize likely causal variants and generate testable functional hypotheses about the underlying mechanisms, a bioinformatics framework was used to query publicly available biological datasets (see Table G). Several databases are available for the functional characterization of putative disease causing loci such as Haploreg [1] (maintained by the Broad Institute), and the University of California, Santa Cruz (UCSC) genome browser [2]. Annotation of non-protein-coding regions operates under the hypothesis that trait-associated alleles exert their effects by influencing transcriptional levels through multiple regulatory mechanisms. Haploreg is useful for an initial survey of a large number of correlated variants comprising GWAS loci for regulatory evidence such as DNaseI Hypersensitivity (DHS), transcription factor binding sites,

histone modifications, eQTLs, protein-binding motif analysis, and evolutionarily conserved regions. This database extracts regulatory information from ENCODE and NIH Roadmap, and compiles a large transcription factor (TF) motif library of position weight matrices (PWMs) from TRANSFAC and JASPAR. These datasets are then integrated with known variants from the 1000 Genomes Phase I populations. After a permissive initial screen for variants with functional evidence from any of the biological datasets within HaploReg, the UCSC genome browser was used for a deeper interrogation and visualization of potential causal variants.

SUPPLEMENTAL FIGURES

Figure A. Q-Q plots of Hispanic study-specific results for systolic and diastolic blood pressure

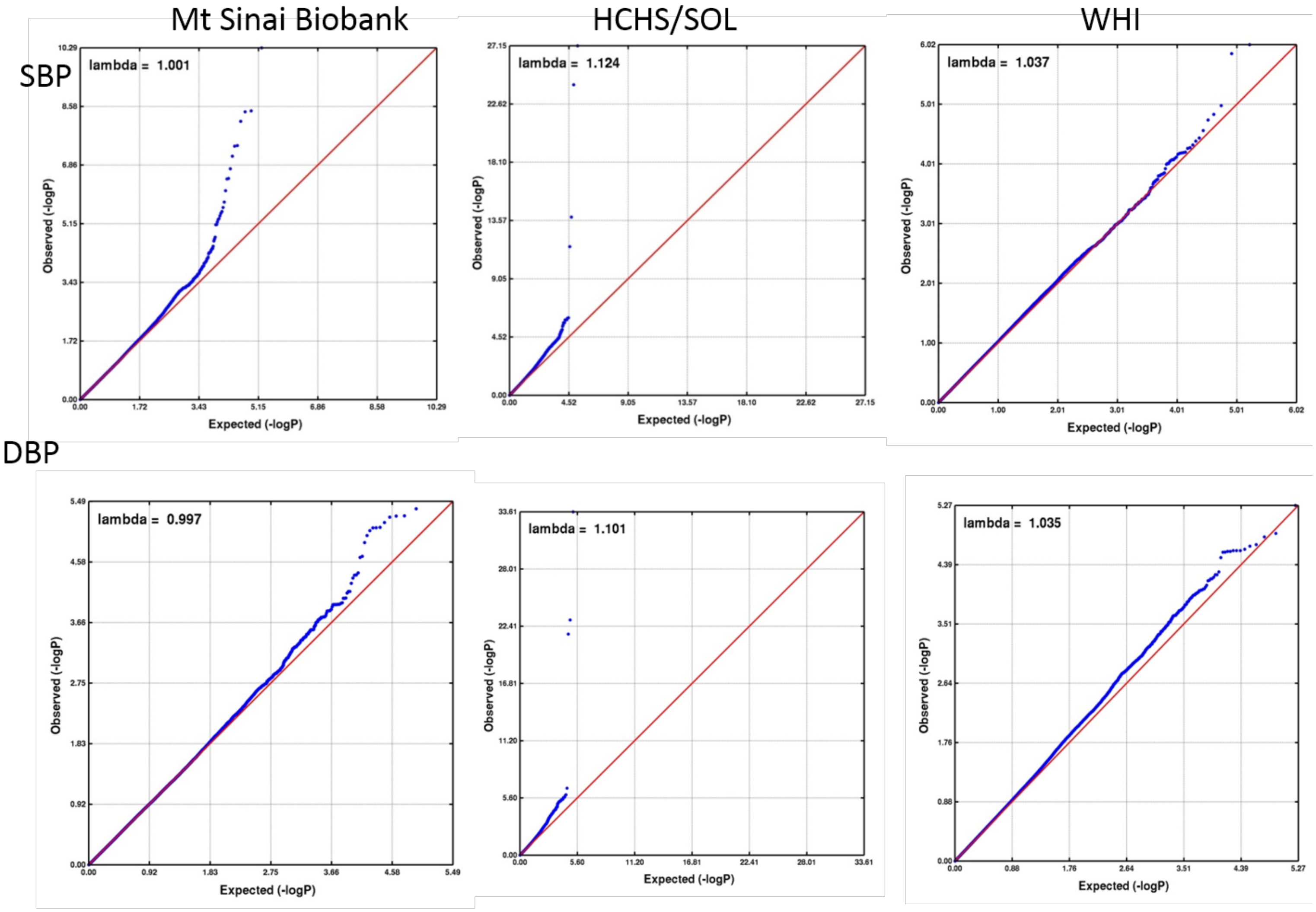

Figure B. Regional plots for *SH2B3* (A) and *TRAFD1* (B) loci for diastolic BP in Hispanics

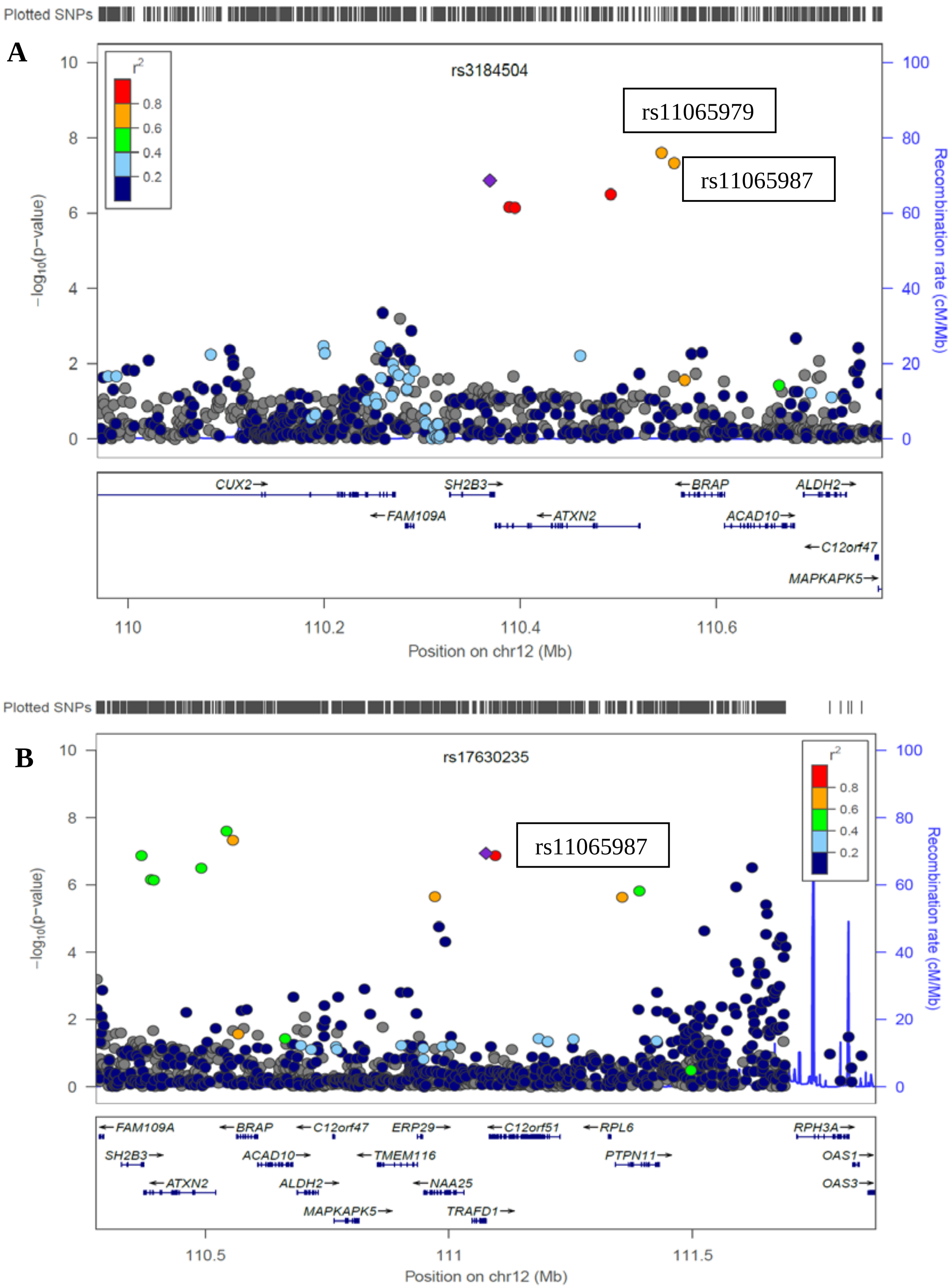

In panel A, the plotted SNP (purple diamond) is a missense variant in high LD with other significant variants in the region. Other SNPs in Table A are also shown.

Figure C. Fine-mapping of the *MTHFR/CLCN6* region in African Americans for diastolic blood pressure

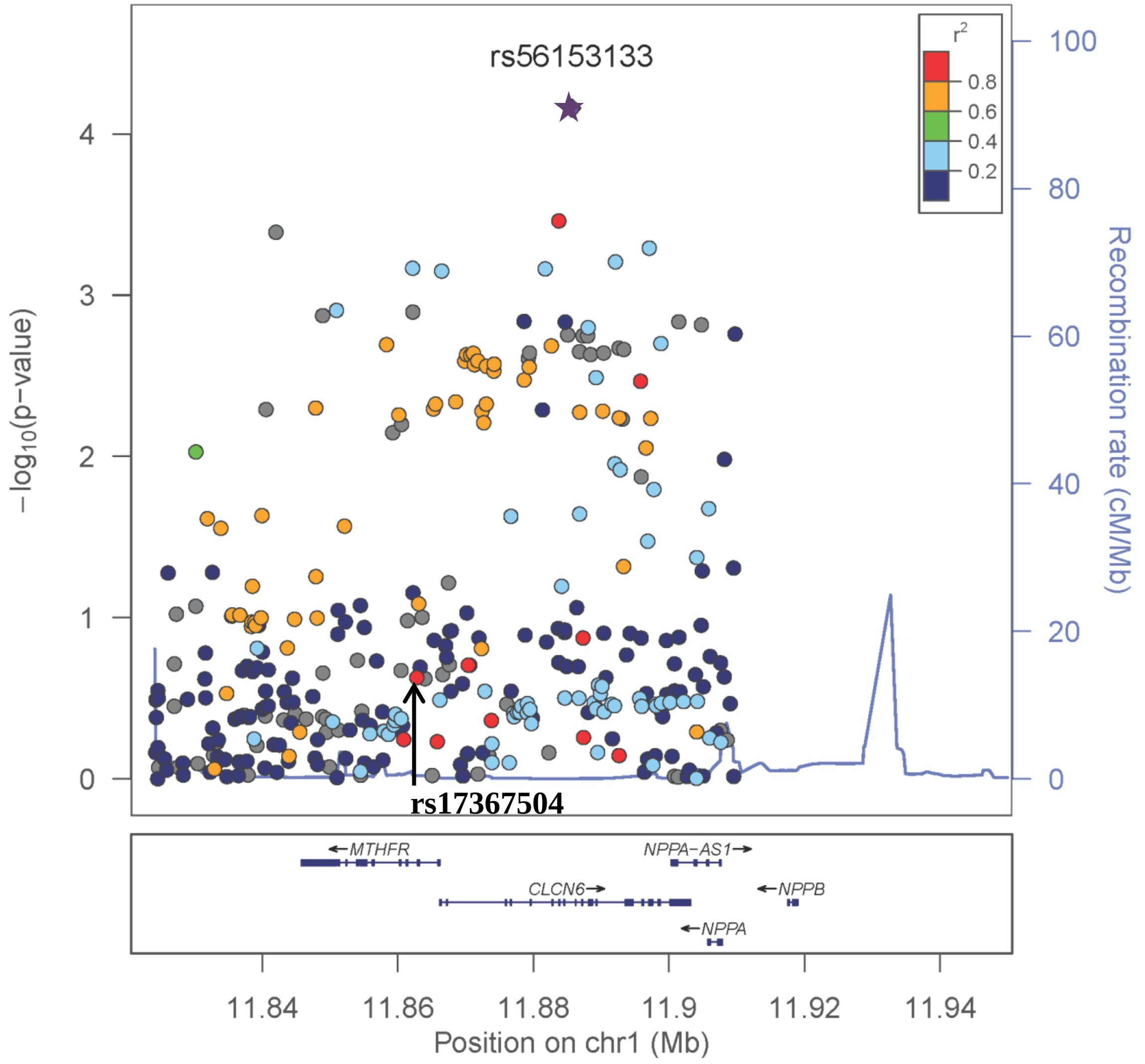

Regional plot of the *MTHFR*/*CLCN6* region in African American samples using LD based on the 1000G European ancestry sample (EUR). The most significant SNP at this locus, rs56153133 (*CLCN6*, purple star), is in high LD with the GWAS SNP rs17367504 (*MTHFR*) in 1000G EUR. However, this SNP was not significantly associated with diastolic BP in PAGE African Americans, suggesting that it may not be the causal variant (if it is assumed that the causal variant is common to multiple ancestral groups).

Figure D. Fine-mapping regional plots of 15q26.1 SBP Locus in African Americans

A. European Ancestry LD (Hg19 1000G EUR) **B. African Ancestry LD (Hg19 1000G AFR)**

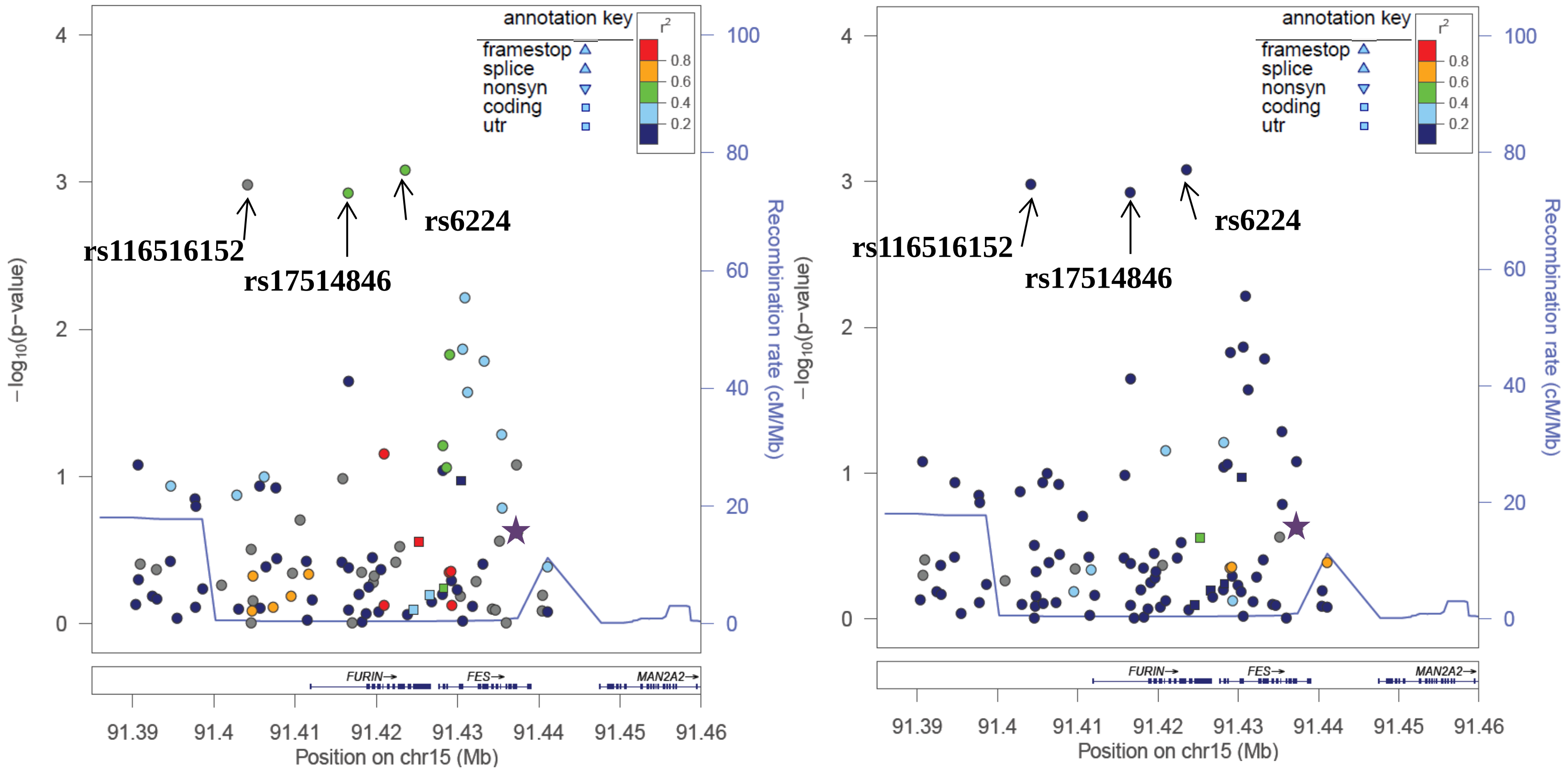

SNPs in both Locus Zoom plots reflect AA p-values. LD between the previously reported GWAS SNP, rs2521501/*FES* (purple star) and other SNPs is shown based on European (panel A) and African (plot B) ancestries. Rs2521501/*FES* is in high LD in 1000G EUR (r^2>0.6) with several SNPs shown in red and orange (panel A), which may make it difficult to know which SNP is driving the signal in EUR. LD across the region is reduced in AA and rs2521501/*FES* is in modest LD with only a few SNPs in AA (r^2>0.4) and none are even nominally significant in AA (panel B). In AA, the best markers in this region are the *FURIN* intronic SNPs, rs6224 and rs17514846, and rs116516152 (7.7kb 5' of *FURIN*); none are strongly correlated with the GWAS SNP in AA, which could suggest that rs2521501/*FES* (purple star) is less likely to be the functional SNP.

Figure E. Trans-ethnic results: fine-mapping regional plots of the SBP *HOTTIP* Locus

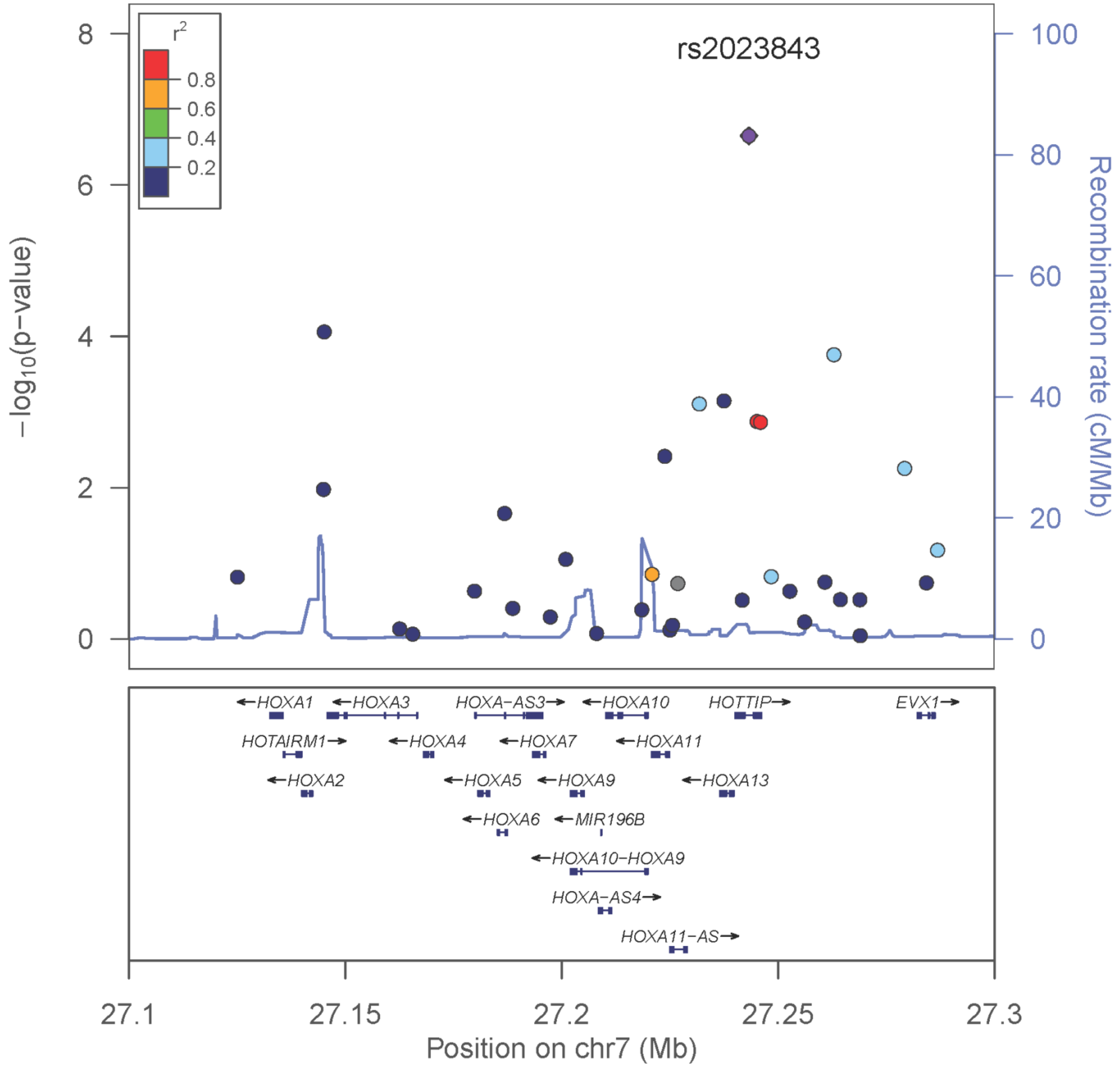

Plot of the *HOTTIP* region using p-values from the trans-ethnic analysis (fixed effects model) and LD based on the 1000G EUR sample. The most significant SNP at this locus, rs2023843 (*HOTTIP*/intron, purple star), is in high LD with other *HOTTIP* SNPs and in modest LD with a few neighbouring SNPs in *EVX1* and the *HOXA* genes. The GWAS SNPs previously reported in African ancestry samples (rs17428471, rs17471520, and rs11564022) located downstream from *EVX1* were not available on the Metabochip, and are not shown. However, LD between rs2023843 and a) rs17428471 or b) rs17471520 is low in both 1000G EUR and AFR, $r^2<0.03$.

LD between rs2023843 and rs11564022 is higher in 1000G AFR, $r^2=0.27$; it is unclear whether these two SNPs reflect the same signal.

Figure F. Trans-ethnic results: fine mapping regional plots of the SBP *FGF5* Locus

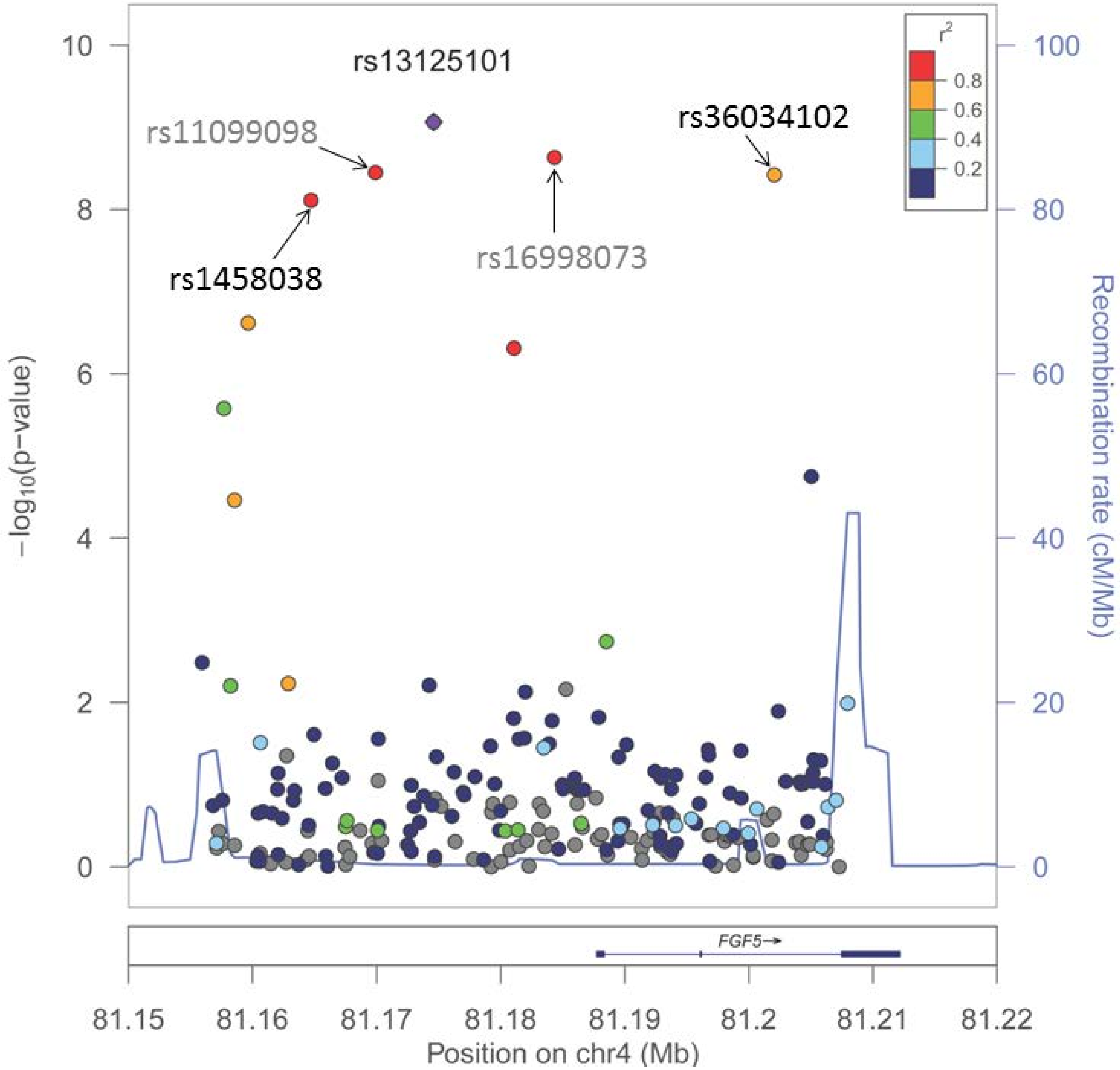

Regional plot of the *FGF5* region using p-values from the trans-ethnic analysis (fixed effects model) and LD based on the 1000G EUR sample. The most significant trans-ethnic SNP at this locus, rs13125101 (purple diamond), is in high LD with the prior GWAS SNPs rs16998073 and rs11099098 (shown in grey text), and with rs1458038, the top SNP in PAGE Hispanics. It is also in high LD with a novel intronic *FGF5* SNP, rs36034102 and a few neighbouring SNPs in the 5`region of *FGF5*.

SUPPLEMENTAL METHODS A

Study Description and Blood Pressure Measurements

PAGE and FBPP Studies

The PAGE consortium includes Hispanic participants from the Hispanic Community Health Study/Study of Latinos (HCHS/SOL, up to 11,653), Women's Health Initiative (WHI, n=5,155) and the Mount Sinai BioMe Biobank (n=2,898). PAGE African Americans were participants from the Atherosclerosis Risk in Communities study (ARIC, n=3,340), the Coronary Artery Risk Development in Young Adults (CARDIA, n=1,797), and WHI (n=11,800). African Americans from the FBPP HyperGEN Study (n=1,245) and the GenNet Study (n=562) were included in the discovery. HyperGen and GenNet are family-based studies ascertained for hypertension, while the remaining studies were population-based studies of unrelated individuals. Adjustment for global ancestry was included in each race/study-specific model using principal components (PCs). The number of PCs used varied by study, but all Hispanic or African American models included at least the first three PCs. The specific number of PCs (over 3) used in a particular study was determined based on prior experience with population stratification adjustment in the study.

ARIC is a multi-center cohort of predominantly white and African Americans [5]. ARIC recruited 15,792 individuals (of which 4,266 are African American) aged 45-64 years from four communities in Forsyth County, N.C., Jackson, M.S., Minneapolis, M.N., and Washington County, M.D. for a baseline examination in 1987-1989, with four follow-up examinations and an examination through 2011-2013. The data used in this study are from the first visit in 1987-1989. A detailed study protocol is available on the ARIC study website (https://urldefense.proofpoint.com/v2/url?u=https-3A__www2.cscc.unc.edu_aric_&d=CwIFAg&c=Zoipt4Nmcnjorr_6TBHi1A&r=iBSSe3ANUkj

PpNQzMcRsTV24Jb8Fi6V1PRcIs4e6qNs&m=fAzniVCF6TuI11xOGNiuKdgD6XsjUMwaVKUfH7TmNuE&s=TAUBClZcTHR2hEvcr9yMo6mtYrMqXbCG_Vlu4oFDSDo&e). BP was measured using a standardized Hawksley random-zero mercury column sphygmomanometer with participants in a sitting position after a resting period of 5 minutes. The size of the cuff was chosen according to the arm circumference. Three sequential recordings for systolic and diastolic blood pressure were obtained; the mean of the last two measurements was used in this analysis. BP lowering medication use was recorded from the medication history.

WHI is a prospective study investigating post-menopausal women's health [6, 7]. A total of 161, 808 women aged 50–79 years old were recruited from 40 U.S. clinical centers between 1993 and 1998 to participate in an observational study and clinical trials. Socio-demographic characteristics, lifestyle factors, medical history, medication use and physical measures of height, weight, and blood pressure, were collected at the baseline visit. BP was measured by certified staff using standardized procedures and instruments. Two BP measures were recorded after 5 minutes rest using a mercury sphygmomanometer, and the average of the two measurements was used in analyses. Appropriate cuff bladder size was determined at each visit based on arm circumference. Diastolic BP was taken from the phase V Korotkoff measures. A total of 5,155 Hispanics and 11,653 African American from WHI with genetic data were included in this analysis.

CARDIA is a population based, prospective cohort examining the development and determinants of clinical and subclinical cardiovascular disease and its risk factors, which recruited 5,115 European Americans (EA) and African American between 18 and 30 years old (52% African American and 55% women) in Birmingham, AL; Chicago, IL; Minneapolis, MN; and Oakland, CA [8]. Baseline measurements were repeated, and additional measurements

performed, at Years 2, 5, 7, 10, 15, and 20. The current analysis included data measured at Year 0 (1985-1986) and only African Americans. Seated BP was measured on the right arm following five minutes rest using a random-zero sphygmomanometer. SBP and DBP were recorded as Phase I and Phase V Korotkoff sounds. Three measurements were taken at 1 minute intervals with the average of the second and third measurements taken for the BP values.

FBPP. The Family Blood Pressure Program (FBPP), composed of four independent networks without overlap in participants (HyperGEN, GENOA, GenNet, and SAPPHIRe), was established to investigate the genetic determinants of high blood pressure (BP) in multiple ethnic groups [9, 10]. Families were ascertained based on higher than normal BP or diagnosed hypertension. The current study includes only genotyped participants of the FBPP HyperGEN and GenNet studies. GenNet sought to address BP as a continuously distributed quantitative phenotype. Non-Hispanic white subjects were recruited from Tecumseh, MI, and African American subjects were recruited from Maywood, Ill. Probands were defined as individuals age 18 to 50 years with BPs in the upper 20% to 25% of the age- and gender-specific BP distribution, and all available first degree relatives irrespective of their BP or hypertension treatment status. HyperGEN (Hypertension Genetic Epidemiology Network) is a multicenter family-based study to research the genetic causes of hypertension and related conditions. HyperGEN recruited African American and non-Hispanic white participants at five field centers: Birmingham AL; Forsyth County, NC; Framingham, MA; Minneapolis, MN; and Salt Lake City, UT. Study participants were recruited as one of three main types of subjects: 1) as part of a hypertensive sibship with at least two siblings diagnosed with hypertension; 2) random subjects, who were age-matched with hypertensive sibs; or 3) unmedicated adult offspring of one or more of the hypertensive siblings. Preference in ascertainment and recruitment was given to hypertensive sibships in which at least

one of the subjects was classified as having severe hypertension. BP was measured using an automated oscillometric BP measurement device with a consistent protocol across the FBPP networks. Two or three BP measures were averaged.

GenNet and HyperGen samples were genotyped on the Metabochip Illumina beadchip array at the University of Texas Health Science Center at Houston and quality controlled at the Johns Hopkins Aravinda Chakravarti Lab. Genotyped individuals included sibships with at least 2 sibs, and both non-Hispanic white and African American individuals. 17 GenNet samples were removed due to poor repeated genotyping quality and 1 GenNet sample was removed due to the gender mismatch. 25 HyperGen subjects failed repeated genotyping and were also excluded. Of 196,725 genotyped SNPs, 20,156 SNPs were monomorphic in all samples genotyped. The agreement between genotype calls for the technical replicates was 0.999. Genotype QC included call rate < 95%, GenTrain score < 0.7, Cluster Separation score < 0.43 (Both GenTrain and Cluster separation scores were selected to be the 5th percentile of the scores distribution), and HWE p-value < 10^{-6}. Nine GenNet samples and 26 HyperGen samples with call rate < 95% were excluded, for a final sample of 1,465 for GenNet and 2,925 for HyperGen. The final data contains 1,230 African American subjects, and 1,695 non-Hispanic white subjects. Only African Americans were included in these analyses. Linear mixed models with kinship coefficients to account for family relationships were used to estimate associations.

HCHS/SOL is a population-based cohort study of 16,415 self-identified Hispanic/Latino individuals aged 18-74 years randomly selected from households in four U.S. field centers (Chicago, IL; Miami, FL; Bronx, NY; San Diego, CA). The cohort includes participants who self-identified themselves as having Hispanic/Latino background, the largest groups being Central American (n=1,730), Cuban (n=2,348), Dominican (n=1,460), Mexican (n=6,471),

Puerto-Rican (n=2,728), and South American (n=1,068). The baseline examination during 2008 and 2011 included a clinical visit with comprehensive biological, behavioral, and socio-demographic assessments [11]. Blood pressures were defined as the average of the second and third of 3 repeat seated measurements obtained after a 5-minute rest (Omron HEM-907 XL). The **Bio*Me* Biobank Program** is an ongoing, prospective, hospital- and outpatient-based population research program operated by The Charles Bronfman Institute for Personalized Medicine (IPM) at Mount Sinai [12]. Bio*Me* is an Electronic Medical Record (EMR)-linked biobank that integrates research data and clinical care information for consented patients at The Mount Sinai Medical Center, which serves diverse local communities of upper Manhattan with broad health disparities. IPM Bio*Me* populations include African Americans 36% Hispanics, 25% African American, 30% EA, and 9% of other ancestry. Information on anthropometrics, demographics, blood pressure values, and use of antihypertensive medication was derived from participants' EMR. For the current analyses, genotype and phenotype data were available on Hispanics.

Replication Study

MESA. We replicated our findings in 2,112 Hispanic and 2,577 African American participants from the Multiethnic study of atherosclerosis (MESA).

MESA is a longitudinal study of subclinical cardiovascular disease and risk factors that predict progression to clinically overt cardiovascular disease or progression of the subclinical disease [13]. Between 2000 and 2002, MESA recruited 6,814 men and women 45 to 84 years of age from Forsyth County, North Carolina; New York City; Baltimore; St. Paul, Minnesota; Chicago; and Los Angeles. Exclusion criteria were clinical cardiovascular disease, weight exceeding 136

kg (300 lb.), pregnancy, and impediment to long-term participation. The MESA Family Study recruited Hispanic and African American participants, generally siblings of MESA participants, using the same inclusion and exclusion criteria as MESA except that clinical cardiovascular disease was permitted. Trained and certified clinic staff collected BP and anthropometric measurements on all MESA participants at baseline. After a 5-minute rest, BP was measured on seated subjects 3 times at 1-minute intervals using the Dinamap PRO 100 automated oscillometric device (Critikon, Tampa, FL) with the back and arm supported. The average of the second and third BP readings was used for analysis.

In addition to the Metabochip, participants in the original MESA cohort, the MESA Family Study and the MESA Air Pollution Study who consented to genetic analyses were genotyped in 2009 using the Affymetrix Human SNP array 6.0. Genotype quality control for these data included exclusion filters on SNP level call rate < 95%, individual level call rate < 95%, and heterozygosity > 53% as described previously [14]. The cleaned genotypic data was deposited with MESA phenotypic data into dbGaP as the MESA SHARe project (study accession phs000209, http://www.ncbi.nlm.nih.gov/projects/gap/cgi-bin/study.cgi?study_id=phs000209.v7.p2); 8,224 consenting individuals (2,685 White, 2,588 non-Hispanic African-American, 2,174 Hispanic, 777 Chinese) were included, with 897,981 SNPs passing study specific quality control (QC). For GWAS, IMPUTE version 2.1.0 was used to perform imputation (chromosomes 1-22) using HapMap Phase I and II - CEU+YRI+CHB+JPT as the reference panel (release #22 - NCBI Build 36 (dbSNP b126)) (only the CEU reference panel was used for imputation in Whites). IMPUTE version 2.2.2 was used to perform imputation for the MESA SHARe participants using the cosmopolitan 1,000 Genomes Phase 1 v3 March 2012 reference set.

SUPPLEMENTAL REFERENCES

1. Newton-Cheh C, Johnson T, Gateva V, et al. Genome-wide association study identifies eight loci associated with blood pressure. *Nature genetics*. 2009;41:666-676
2. Levy D, Ehret GB, Rice K, et al. Genome-wide association study of blood pressure and hypertension. *Nature genetics*. 2009;41:677-687
3. Ehret GB, Munroe PB, Rice KM, et al. Genetic variants in novel pathways influence blood pressure and cardiovascular disease risk. *Nature*. 2011;478:103-109
4. Wain LV, Verwoert GC, O'Reilly PF, et al. Genome-wide association study identifies six new loci influencing pulse pressure and mean arterial pressure. *Nature genetics*. 2011;43:1005-1011
5. The Atherosclerosis Risk in Communities (ARIC) study: Design and objectives. The ARIC investigators. *Am J Epidemiol*. 1989;129:687-702
6. Design of the Women's Health Initiative clinical trial and observational study. The Women's Health Initiative study group. *Control Clin Trials*. 1998;19:61-109
7. Hays J, Hunt JR, Hubbell FA, Anderson GL, Limacher M, Allen C, Rossouw JE. The Women's Health Initiative recruitment methods and results. *Ann Epidemiol*. 2003;13:S18-77
8. Friedman GD, Cutter GR, Donahue RP, Hughes GH, Hulley SB, Jacobs DR, Jr., Liu K, Savage PJ. CARDIA: Study design, recruitment, and some characteristics of the examined subjects. *J Clin Epidemiol*. 1988;41:1105-1116
9. Multi-center genetic study of hypertension: The Family Blood Pressure Program (FBPP). *Hypertension*. 2002;39:3-9
10. Williams RR, Rao DC, Ellison RC, Arnett DK, Heiss G, Oberman A, Eckfeldt JH, Leppert MF, Province MA, Mockrin SC, Hunt SC. NHLBI Family Blood Pressure Program: Methodology and recruitment in the HyperGen network. Hypertension genetic epidemiology network. *Ann Epidemiol*. 2000;10:389-400
11. Lin DY, Tao R, Kalsbeek WD, Zeng D, Gonzalez F, 2nd, Fernandez-Rhodes L, et al. Genetic association analysis under complex survey sampling: the Hispanic Community Health Study/Study of Latinos. Am J Hum Genet. 2014;95(6):675-88.
12. Tayo BO, Teil M, Tong L, Qin H, Khitrov G, Zhang W, Song Q, Gottesman O, Zhu X, Pereira AC, Cooper RS, Bottinger EP. Genetic background of patients from a university medical center in Manhattan: Implications for personalized medicine. *PLoS One*. 2011;6:e19166
13. Bild DE, Bluemke DA, Burke GL, et al. Multi-ethnic Study of Atherosclerosis: Objectives and design. *Am J Epidemiol*. 2002;156:871-881
14. Manichaikul A, Naj AC, Herrington D, Post W, Rich SS, Rodriguez A. Association of *SCARB1* variants with subclinical atherosclerosis and incident cardiovascular disease: the Multi-ethnic Study of Atherosclerosis. *Arterioscler Thromb Vasc Biol*. 2012 Aug;32(8):1991-9.
